# Nursing Progress and Its Development: V. The Nurse-Pupil's First Stage

**Published:** 1918-01-12

**Authors:** 


### THE MATRONS' AND SISTERS' DEPARTMENT.

### NURSING PROGRESS AND ITS DEVELOPMENTS.

### V. The Nurse-Pupil's First Stage.

We have been at some pains in these columns to set forth the curriculum which prevails in the larger British nurse-training schools at the present time, and have already pointed out that the schools which are leading the van of progress are closely linked to the College of Nursing through the adhesion of their matrons. Although the war, by increasing the work and diminishing the number of trained workers available, has made the task twice as difficult as it used to be, the business of training nurses is proceeding unhindered, and if some advantages are lost to war-time probationers, others are afforded them. It will be convenient, in appraising the future work which lies before the College of Nursing, to summarise briefly the training now assumed to be essential to a well-trained nurse.

In her passage from an ignorant novice to a skilled nurse the probationer passes through several well-defined stages. There is first the period of preliminary training before she begins to do any nursing proper. Secondly, the period of pupilage in the wards when she is acquiring nursing skill and some acquaintance with the nature of disease. Then follows a period of more advanced instruction, when she is enlarging her experience and learning how to manage a ward and how to teach others. Lastly, there is a stage in which she gains insight into such departments of hospital work as housekeeping or office work, and perfects herself in some special branch of work, fever, midwifery, massage. In all these stages the College of Nursing will be able to stimulate progress.

It is now very generally admitted that the first period of training is best afforded to the probationer in a preliminary school, either in the hospital or in an adjoining building. Tredegar House, in connection with the London Hospital, and the preliminary schools at Guy's and St. Bartholomew's are fine models of what such an establishment ought to be, and we have lately published the curriculum and time-table of the Nightingale Preliminary School, which is worthy of imitation. There are still many training-schools where, as at King's College Hospital, the opinion prevails that the probationer loses time by beginning her term of instruction elsewhere than in the wards. But however advantageous the plan of consecutive teaching may prove under a well-thought-out system, it remains undeniably true that the probationer is not the only person to be considered in this matter. There is the well-being of the ward, the time-scheme of the sisters and staff nurses, the tranquillity of the patients, the convenience of the medical staff. Any and all of these are apt to be imperilled by the incursion of untrained women into a sphere for which they are wholly unprepared. For this reason, and also because the miserable scramble in which the new probationer usually passes her first weeks forms a very undignified entry into the profession, it may be assumed that, except under very special conditions, regular preliminary training will come more and more to be reckoned an essential part of the probationer's course.

In London and in many other large towns, where the demand for prepared probationers is continuous, there would seem to be an opening for central preliminary training-schools which could relieve the hospitals of part of the financial and other burdens involved in receiving and testing pupils who desire to take up nursing. To found such a school the active co-operation of the matrons at the head of the local nurse-training schools would, of course, be indispensable. Only through their common membership of a central nursing body like the College could such co-operation be brought about. It is more than probable that the municipal authorities in many towns would favour and agree to subsidise such undertakings. Under good management they would not only prove of value to the hospitals and to the public, but would also prove a much needed stimulus in inducing capable women to embrace the career of a nurse. Should the formation of such centres be found advisable in the near future, it is to the College of Nursing that matrons must look for their establishment. Short of this, and the time may not yet be ripe for the larger scheme, all who are concerned in helping to reduce the "awkward period" in the wards to a minimum look to the College to give counsel on the difficult art of preparing untrained women for their first introduction to a nursing life, and formulate a syllabus for the benefit of training-schools still in the rear.

